# Promoting self-determined Indigenous data governance in Canada: the Métis Health Research and Data Governance Principles

**DOI:** 10.1093/heapro/daaf229

**Published:** 2026-01-13

**Authors:** Robert Henry, Chelsea Gabel, Caroline Tait, Kiera Kowalski, Alexandra Nychuk

**Affiliations:** Department of Indigenous Studies, University of Saskatchewan, 234 Kirk Hall, Saskatoon, Saskatchewan, Canada S7N 5B5; Department of Health, Aging and Society, McMaster University, 235 Kenneth Taylor Hall, Hamilton, Ontario, Canada L8S 4M4; Department of Indigenous Studies, McMaster University, 1811 L.R. Wilson Hall, Hamilton, Ontario, Canada L8S 4M4; Faculty of Social Work, University of Calgary, 302 MacKimmie Hall, Calgary, Alberta, Canada T2N 1N4; Department of Community Health Sciences, Cummings School of Medicine, 3D10, 3280 Hospital Drive NW, Calgary, Alberta, Canada T2N 4Z6; Department of Health, Aging and Society, McMaster University, 235 Kenneth Taylor Hall, Hamilton, Ontario, Canada L8S 4M4; Department of Health, Aging and Society, McMaster University, 235 Kenneth Taylor Hall, Hamilton, Ontario, Canada L8S 4M4

**Keywords:** Indigenous health, data sovereignty, data governance, ethics, self-determination, Métis research principles

## Abstract

Population-level data collection is crucial to advance Indigenous rights and sovereignty but requires localized approaches to develop representative datasets. In Canada, a focus on First Nations research and data governance and principles has led to the underrepresentation of Métis realities and a reliance on data governance models that fail to address their unique cultural, historical, and community-specific needs. “The Saskatchewan Métis Health Research and Data Governance Principles©” were developed to guide Métis research and promote Métis data sovereignty. While these principles share similarities with the First Nations Principles of OCAP®, they emphasize Métis-specific priorities such as capacity building and active engagement with Métis rights holders. These principles provide a framework for Métis health research, ensuring that Métis values and perspectives are embedded throughout the research lifecycle.

## Graphical Abstract

**Graphical Abstract:**
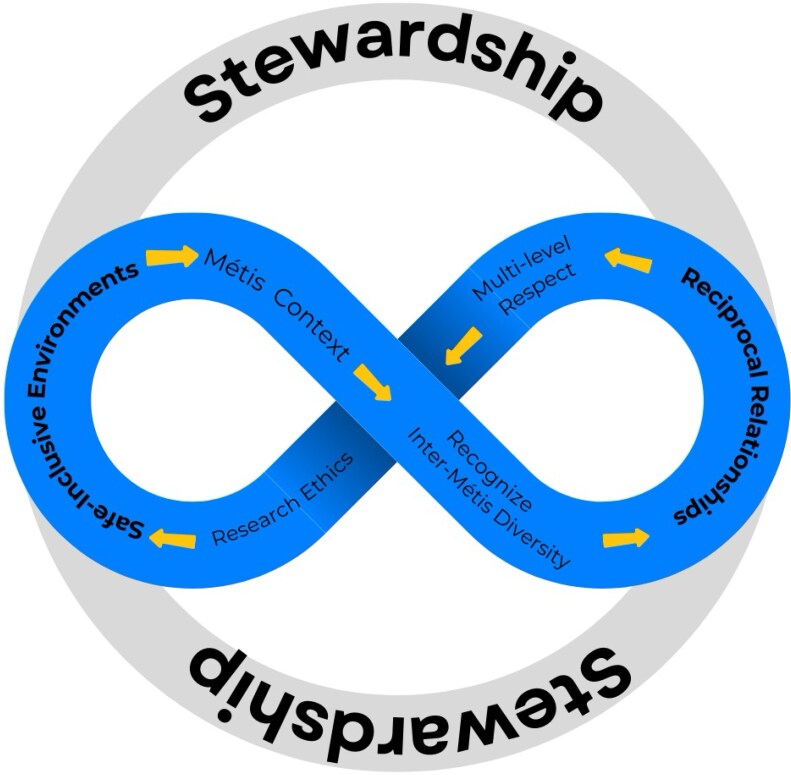
The Saskatchewan Métis Health Research and Data Governance Principles (reciprocal relationships, respect, safe and inclusive environments, diversity, research ethics, and Métis context) guide the relationship between Métis rights holders and their research partners. This relationship is the foundational basis that data stewardship is agreed upon in Métis contexts. Image Copyright 2022 by Dr. Caroline Tait. Adapted with permission.

Contributions to Health Promotion

- Address the need for Métis-specific health research
- Promote Métis control over existing health data
- Improve Métis health and well-being through evidence-based programs and services
- Provide non-Indigenous researchers with culturally appropriate tools to collaborate with Métis people on health research projects

## Introduction

In Canada, the focus on Indigenous (First Nations, Inuit, and Métis) data rights, sovereignty, and governance has primarily been understood and applied through a First Nations lens (Tait and Henry 2022). While First Nations data principles and strategies have been instrumental in the advancement of Indigenous research, their widespread application beyond their intended scope fails to recognize distinction-based and localized approaches to research and data governance. Evans *et al.* (2012) argued over a decade ago that “the paucity of Métis research will continue as long as research guidelines fail to account for the distinctiveness of Métis realities. There is a likelihood that Métis realities will be assumed to coincide with that of other [Indigenous] communities which have been the focus of extensive research and thus have come to represent ‘[Indigeneity]’ in Canada” (p. 56). Thus, Métis people, who are one of the three constitutionally recognized Indigenous peoples in Canada, are significantly behind in research and policy environments and remain stuck in data dependency. Métis peoples and communities require localized approaches to research that build capacity for the development of robust datasets, specifically for health and well-being. The “Saskatchewan Métis Health Research and Data Governance Principles©” were designed to recognize the distinct history and localized cultures of Métis peoples to support the advancement of Métis research interests across Canada. The Métis principles share many similarities with the First Nations Principles of OCAP® and the now defunct National Aboriginal Health Organization’s (NAHO) “Principles of Métis Ethical Research”; however, there are significant differences that reflect contemporary Métis research interests, including capacity to engage in research. Specifically, the Métis principles aim to highlight the importance of Métis values and perspectives beyond just data governance to include collaboration with Métis rights holders at every step of the research lifecycle. This paper seeks to position Métis-specific data governance and principles within both international and domestic discussions on Indigenous data sovereignty (IDS). We will introduce the “Saskatchewan Métis Health Research and Data Governance Principles©” and provide an overview on their applicability to guide research engagement within Métis health and well-being-specific research contexts.

## Indigenous data rights

Data are considered one of the most valuable resources across both private and public sectors, as it fuels innovation and capital. Despite the global push for large datasets among the open data movement, marginalized peoples remain on the periphery of population-level data collection (Walter *et al*. 2025). For Indigenous peoples, these decisions continue to reproduce settler colonialism as they neglect distinction-based data rights (Wolfe 2006, Kukutai and Walter 2015, Carroll *et al*. 2019, Rainie *et al*. 2019). The United Nations Declaration on the Rights of Indigenous Peoples acknowledges the distinct, inherent, and collective data rights of Indigenous peoples that extend beyond individual rights and are not shared by other ethnic or minority groups (Banerjee 2003, United Nations 2018, Rainie *et al*. 2019). The United Nations states that “Governments and corporations [must] recognise the sovereignty of Indigenous peoples over data that are about them or collected from them, and which pertain to Indigenous [P]eoples, knowledge systems, customs or territories, by always including formalised Indigenous developed principles, a focus on Indigenous leadership and mechanisms of accountability” (United Nations 2018, para 9).

Indigenous data are defined as “data generated by Indigenous Peoples, as well as by governments and other institutions, on and about Indigenous Peoples and territories, as well as information about Indigenous communities and the individuals, Indigenous and non-Indigenous, that live within” (Carroll *et al*. 2020, p. 3). Indigenous peoples and nations require data to improve their health and well-being, but many remain unable to access and create localized specific data for their needs (Kukutai and Walter 2015, Carroll *et al*. 2019, Rainie *et al*. 2019). Without opportunity and accurate representation, Indigenous peoples cannot engage in effective advocacy and decision-making required to address health inequities, holding them in a state of data dependency (Kukutai and Walter 2015, Andersen 2016, Carroll *et al*. 2019, 2020, Rainie *et al*. 2019).

**Figure 1:**
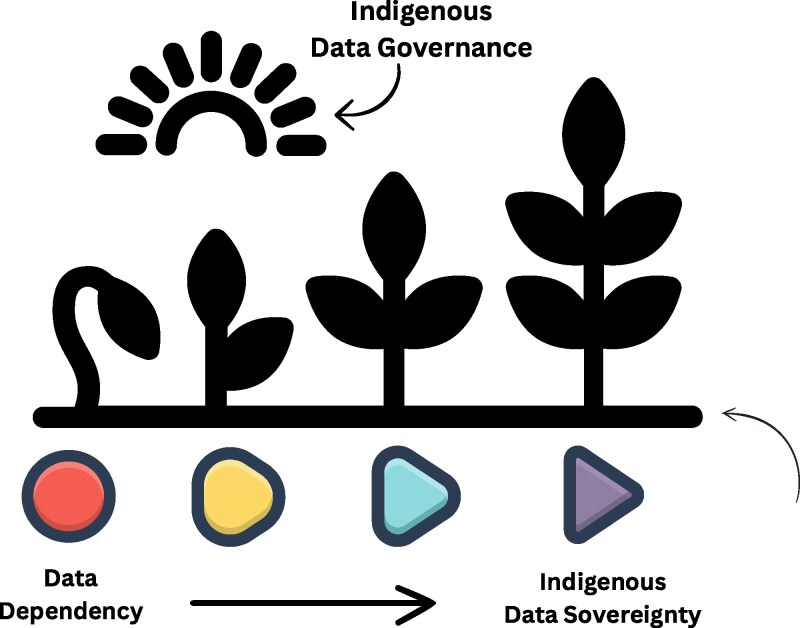
Depicts the role that Indigenous Data Governance plays in advancing data dependency to IDS.

“Data dependency” in the case of Indigenous peoples occurs when outside agencies, colonial nation states, or other bodies collect and hold data about Indigenous peoples or nations, or when there is a lack of accurate data conducted by and for Indigenous peoples or nations (Carroll *et al*. 2019). To combat data dependency, many Indigenous nations are building capacity toward IDS as a method to facilitate self-determination and intergenerational health and well-being (Cormack *et al*. 2019, Kukutai 2023). IDS is described as the right of “each [Indigenous Nation] to control the collection, ownership, and application of its own data” (Rainie *et al*. 2017, p. 1). In order for IDS to exist, strong “Indigenous data governance” must be established which consists of two interrelated elements (i) Data for Governance and (ii) Governance of Data (Smith 2016, Carroll *et al*. 2019). Data for Governance refers to the accessibility, accuracy, relevancy, and timeliness for Indigenous nations to create evidence-based policies, whereas Governance of Data refers to protection and control of Indigenous data, including policies and procedures for Indigenous data that are held by outside agencies, nations, or bodies (Fig. 1).

## Global trends in Indigenous data governance

The altruistic intentions that have informed the global open data movement are guided by blanket policies designed by the FAIR (Findable, Accessible, Interoperable, Reusable) principles, which call for scientific data management and stewardship to support innovation, discovery, and decision-making for the betterment of society (Wilkinson *et al*. 2016). Many Indigenous scholars from across the globe have voiced their concerns about Indigenous peoples’ and nations’ ability to exercise inherent and collective rights using the FAIR framework within big data, open data, and open science environments (Carroll *et al*. 2021). In response to the FAIR principles, the CARE (Collective Benefit, Authority to Control, Responsibility, Ethics) principles were created by the CANZUS (Canada, Australia, Aotearoa/New Zealand, and the USA) IDS networks which are comprised of Indigenous scholars, practitioners, and activists (Walter *et al*. 2025). The CARE principles aim to unearth the structural disadvantages, historical contexts, and power dynamics that inhibit Indigenous peoples from accessing their data (Carroll *et al*. 2021). They are intended to compliment the FAIR principles by encouraging the open data movement to recognize the crucial role of data in advancing Indigenous innovation and self-determination.

Indigenous peoples in all four CANZUS countries continue to implement the FAIR and CARE principles through their IDS Networks. Most IDS networks have created specific data charters, principles, and/or guidelines which are informed by their local context and knowledge including their unique relationship to the settler colonial government (Kukutai 2025). Common features among the CANZUS IDS networks include (i) a focus on self-determination and intergenerational well-being, (ii) recognition of data as a valued cultural resource, (iii) an emphasis on collective data rights, and (iv) prioritization of Indigenous values as the basis for good data governance (Kukutai 2025). However, not all Indigenous nations’ interests are represented in the IDS networks. For example, the Canadian IDS network is directly tied to the First Nations Information Governance Centre (FNIGC), which protects the interests of First Nations data. The other two constitutionally recognized Indigenous peoples in Canada, the Inuit and Métis, are not represented under this network (Kukutai 2025) primarily due to the lack of data infrastructure.

### Data architecture

Conversations about Indigenous data remain primarily associated with governance and sovereignty and rarely explicitly extend to data architecture. The Data Management Association (DAMA) defines data architecture as “an organized arrangement of component elements intended to optimize the function, performance, feasibility, cost, and aesthetics of an overall structure or system” (Data Management Association 2017, p. 97). Well-conceived data architecture provides the potential for data stewards to gain a greater understanding across systems, ensure compliance with data policies and regulations, and enable decision-making (Leone 2021). The current data ecosystem in Canada continues to perpetuate Indigenous data dependency, obfuscating not only broader Indigenous data rights but also the acknowledgment of the distinctiveness and self-determined priorities both across and within FNIM in Canada. Leone (2021) asserts that Indigenous data architecture centers on three components: (i) relationships and accountability, which includes the relationships between and within FNIM organizations, and any actor that collects FNIM data, (ii) capacity, which refers to the ability for FNIM to manage their data and acquire technical capacity through expertise and literacy in data, and (iii) control, which refers to the need for control beyond data architecture to other areas such as data integration, data security, and data governance. Good data architecture accounts for the reality that these components must be adaptable and dependent on balanced stewardship between stakeholders. Such arrangements are established through well-maintained relationships and ongoing accountability, as data ecosystems constantly change as technology, linkages, and politics remain in a state of flux. For example, high-level governance (national/regional level) accounts for specific needs and priorities of the smaller, more localized forms of governance. “The Saskatchewan Métis Health Research and Data Governance Principles©” described below directly address relationships and accountability, offering a framework to support the development of robust data architecture while helping Métis governing bodies and nations advance their data governance efforts.

## The state of Métis data

Métis, one of the three constitutionally recognized Indigenous peoples in Canada, emerged as a distinct Indigenous people and nation across the Northwest during the 18th century prior to the establishment of Canada (MacDougall 2010, Andersen 2016, Teillet 2019, Bartel 2024). The struggle to be constitutionally recognized was a long and arduous battle for the Métis that continues today. A series of cases (R. v. Powley 2003, Manitoba Métis Federation Inc v. Canada 2013, Daniels v. Canada 2016) heard at the Supreme Court of Canada in the early 2000s defined the Métis’ distinct rights and the government’s responsibility to them as an Indigenous people; however, many of these commitments have yet to be operationalized by the federal government (Kermoal and Andersen 2021). For example, the Métis are the only recognized Indigenous peoples whose health remains a provincial fiduciary responsibility, whereas status First Nations and Inuit fall under the federal noninsured health benefits program administered by Indigenous Services Canada (Gabel *et al*. 2017).

Métis scholars Drake and Gaudry (2016) argue that the Métis’ ongoing struggle for recognition lags 10–15 years behind that of First Nations in Canada, which is certainly true within the Métis data governance landscape. The need for localized approaches to Métis data governance was highlighted during the COVID-19 pandemic when health data were crucial for decision-making and citizen safety. Both the federal and provincial health authorities were collecting data on Indigenous people that included representation from Métis citizens, but there were no mechanisms in the reporting to indicate Métis citizenship or a health data linkage to streamline the information to the Métis Governing Bodies, rendering them statistically invisible. NHIB was reporting on the status of COVID-19, but only for First Nations and Inuit through pre-existing data linkages (Richmond *et al*. 2020, Ruckstuhl 2022). Not only did this violate Métis Nations and Governing Bodies’ data rights but also obstructed Métis political leaders from having accurate data to make decisions pertaining to their citizens’ health.

Among the struggle for recognition, response mobility continues to create a growing concern within the Métis nation, as many people continue to self-identify as Métis without demonstrating connection to the historic and contemporary Métis governing bodies, which include the Manitoba Métis Federation, Métis Nation of Alberta, Métis Nation-Saskatchewan (MN-S), Métis Nation of British Columbia, and Métis Nation Ontario (Gaudry and Andersen 2016, Gaudry and Leroux 2017, O’Donnell and LaPointe 2019). The census data collection in Canada has been particularly misleading when it comes to the dissemination of Métis-specific data (Andersen 2016). Between 2006 and 2016, only one question was included in the national long form census that pertained to Métis identity and was part of a more general question asking “Is this person an Aboriginal [Indigenous] person, that is, First Nations (North American Indian), Métis or Inuk (Inuit)” (Turner 2019, para 18). In 2016, the Government of Canada claimed Métis were one of the fastest-growing populations in the country; however, Métis governing bodies and nations’ citizenship registries did not demonstrate the same level of growth (Andersen 2016). This dataset was alarmingly inaccurate as a growing number of individuals in Canada self-identified as Métis but did not possess Métis citizenship (Gaudry and Leroux 2017). In the subsequent 2021 census, the Canadian government attempted to rectify their discrepancy by adding an additional question to clarify if the individuals who self-identified as Métis were members of a Métis organization or citizens of a Métis government (Statistics Canada 2022). The data revealed that only one-third of those individuals who self-identified as being Métis also reported having citizenship or membership with a legitimate Métis organization or settlement (Statistics Canada 2022). The Canadian government’s willingness to overlook Métis rights within the census shows how Métis are lacking both data for governance and governance of data (Andersen 2014, Tait and Henry 2022). As the Métis continue to gain recognition as a people, and therefore self-determination, there is a need for a robust and distinct Métis data strategy to inform advocacy, policy, and decision-making efforts.

## The need for distinction-based research and data governance

The 1996 Royal Commission on Aboriginal Peoples (RCAP) Final Report was the catalyst for Indigenous data governance in Canada. The commission was established in 1991 to investigate the relationships between Indigenous peoples, the Government of Canada, and Canadian society as a whole. The report recommended that a working group of First Nations, Inuit, and Métis peoples be established to “1) collaborate with [Indigenous] governments and organizations to establish and update statistical databases and 2) promote data governance strategies across nations and communities for collecting and analyzing data” (First Nations Information Governance Centre 2024, para 5). Rather than a working group of First Nations, Inuit, and Métis, a National Steering Committee of First Nations and Inuit was established to roll out the First Nations and Inuit Regional Longitudinal Health Survey as a result of the RCAP recommendations. Following the development and execution of this survey, First Nations health leaders further lobbied the federal government for control of on-reserve health data, which led to the development of the OCAP® principles (Ownership, Control, Access, and Procession) that are now governed by the FNIGC (Tait and Henry 2022, First Nations Information Governance Centre 2024). The OCAP® principles have become the most widely known and cited Indigenous research guidelines throughout the Indigenous research landscape in Canada. They outline how First Nations’ “data and information will be collected, protected, used or shared” (First Nations Information Governance Centre 2024). In 2015, the FNIGC launched the Fundamentals of OCAP® Training dedicated to implementing the principles in research and data management. The training led to the widespread use of the principles by Indigenous and non-Indigenous researchers and organizations from First Nations, Inuit, and Métis communities, across various research and data environments.

The OCAP® principles have highlighted the need for IDS and created visibility for localized efforts to Indigenous data governance. However, their application across various Indigenous research contexts, including those that pertain to Inuit and Métis peoples violates the OCAP® trademark. The OCAP® principles cannot technically be used outside of a First Nations context, as they are collectively owned by all First Nations people under the stewardship of the FNIGC (First Nations Information Governance Centre 2024). The FNIGC pursued copyright to protect the principles from misuse, misapplication, and improper interpretation (First Nations Information Governance Centre 2024). The broad use of the principles fails to recognize the distinct histories and diversity of Indigenous peoples in Canada, which inherently inform localized approaches to data sovereignty and governance.

Since 1998, Inuit Tapiriit Kanatami (ITK) has been recognized as the national representative body for the advancement and protection of Inuit rights in Canada (Inuit Tapiriit Kanatami 2018). ITK has published several resources explaining how to effectively engage in Inuit self-determined research, which until recently defaulted to the FNIGC’s OCAP® principles. In 2018, the Qanuippitaa? National Inuit Health Survey was developed by Inuit from across Canada, in collaboration with ITK. The survey ensures that data accurately reflects Inuit life by adapting data collection methods and ensuring that data collectors are from the regions they serve. In the same year, ITK also released its National Inuit Strategy on Research which emphasizes the need for Inuit leadership in shaping research agendas and outlines actionable steps to address the high prevalence of non-Inuit researchers working in the North (Inuit Tapiriit Kanatami 2018). While the strategy does not explicitly call for the adoption of the First Nations Principles of OCAP®, it highlights the importance of Inuit involvement in the governance of research within Inuit Nunangat to ensure Inuit access, ownership, and control over data and information. Most recently, in 2022, the Government of Canada, specifically, Canadian Institutes of Health Research (CIHR), committed $6.4 million in directed funding to establish an Inuit Research Network. This funding supports the four Inuit regions and their respective land claims organizations—Inuvialuit Regional Corporation, Nunavut Tunngavik Incorporated, Makivik Corporation, and the Nunatsiavut Government—to guide research that strengthens Inuit health.

**Figure 2:**
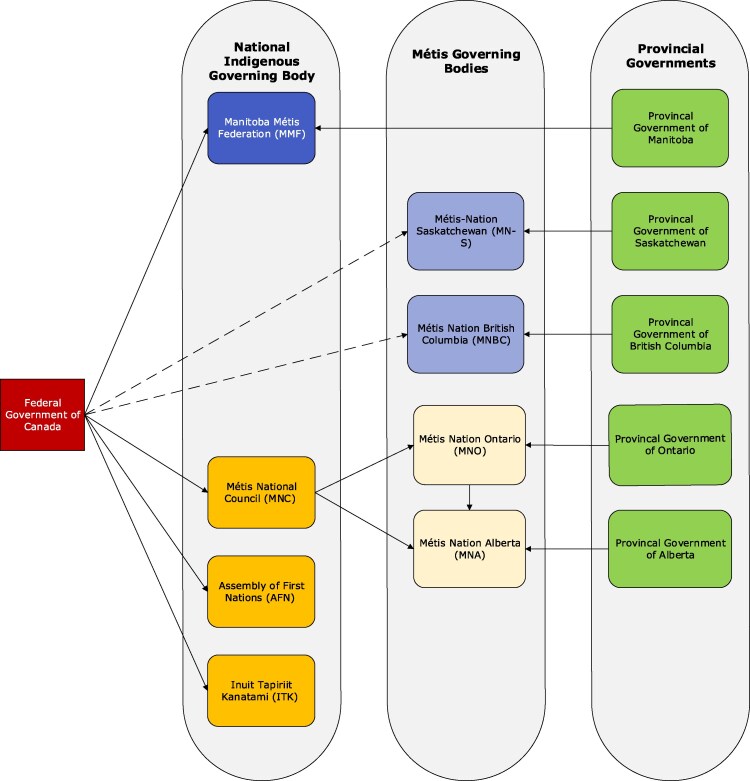
Depicts the current climate of recognized Métis governmental bodies and nations and their respective relationships to both Federal and Provincial governments in Canada.

The Métis are overlooked within health research environments. There are at least three reasons for this. First, with a few notable exceptions (such as the Métis settlements in Alberta), Métis communities have not received the ongoing funding necessary to develop the infrastructure found in many First Nations communities or larger regional organizations. While Métis local, provincial, and national organizations do exist, their stability tends to diminish as the scale increases. Administrative coverage within individual Métis communities is often lacking, and even when administrative structures are in place, these communities receive far less funding than First Nation organizations, whether regional or community specific. Communities without formal infrastructure are harder to locate administratively, making it more difficult—and less likely—that they will apply for available funding (Evans *et al*. 2012). Second, place-based definitions of community typically view it as singular and localized (for instance, a First Nations reserve). However, Métis peoples, unlike First Nations, do not live on reserves and more often than not, live in urban centers and belong to multiple communities simultaneously (Evans *et al*. 2012). Thirdly, the contemporary representation of the Métis at the national level continues to be a point of contention. From 1983 to 2021 the Métis National Council (MNC) represented its provincially based affiliates in Manitoba (the Manitoba Métis Federation); Alberta (the Métis Nation of Alberta); Saskatchewan (MN-S), and later Ontario (Métis Nation of Ontario) and British Columbia (the Métis Nation of British Columbia; Weinstein 2007). During this time the MNC was a collective voice for the Métis, engaging in various nation-to-nation relationships with different levels of government. However, in 2021, the Manitoba Métis Federation announced its decision to leave the MNC due to membership disputes involving the Métis Nation of Ontario (Manitoba Métis Federation 2021). Debate about membership, including who is and who is not Métis, has caused divisions among the various provincial Métis political bodies. Since MMF’s decision, the MN-S and the Métis Nation of British Columbia have followed suit, leaving the MNC in 2024 (Métis Nation British Columbia 2024, Métis Nation-Saskatchewan 2024; see Fig. 2).

Collectively, these tensions have led to a lack of recognition in Canadian health research environments. The CIHR, the federal funding agency responsible for health research in Canada provided <5% of the total dollars awarded to Indigenous health research to Métis-specific health research, despite the Métis constituting over 31.5% of the Indigenous population in Canada (Canadian Institutes of Health Research 2024, Nychuk 2024). As such, there are large gaps in the understanding Métis health and well-being, as well as how to develop ethical research partnerships and agreements to ensure sovereignty and governance over Métis health and well-being research information.

## The Métis Health Research and Data Governance Principles

The “Saskatchewan Métis Health Research and Data Governance Principles©” were created through a partnership between MN-S and Métis citizens Dr Caroline Tait and Dr Robert Henry for Métis rights holders in their research and data sharing partnerships with researchers and government institutions (Tait and Henry 2022). They reflect the values and priorities of MN-S citizens but are applicable to diverse Métis populations, organizations, and communities and can be adapted for local, regional, provincial, and national contexts (Tait and Henry 2022). Table 1 highlights the diversity of Métis governments across Canada and the language used to describe the different levels within them. Furthermore, the “Saskatchewan Métis Health Research and Data Governance Principles©” represent a snapshot in time and are designed to be updated as Métis data governance needs evolve. They do not intend to overshadow the rights of each of the Métis governing bodies to develop their own internal ethics processes but act as a framework for undertaking research involving Métis peoples. This is critical, as the Métis is not a homogeneous group and have diverse socio-political experiences and independent government structures.

**Table 1:** Describes the different language used by Métis governing bodies and nations to describe the varying levels of their respective governance structure.

Governing body/nation	Regional level	Grassroots/community level
Manitoba Métis Federation	Region	Local
Métis Nations-Saskatchewan	Region	Local
Métis Nation-Alberta	Territories	District
Métis Settlements General Council (found in Alberta)	–	Settlements
Métis Nation British Columbia	Region	Chartered communities
Métis Nation Ontario	Region	Chartered communities

The principles are derived from the first Métis-specific research engagement guide, the “Principles of Métis Ethical Research,” developed by the Métis Centre of the NAHO (MC-NAHO) in 2010. This guide was created through a “think tank” that included Métis researchers, students, and organizations (Tait and Henry 2022). MC-NAHO designed the principles for internal use but also envisioned that they would be utilized by researchers engaging in research with Métis communities. The six principles created during the think tank were reciprocal relationships, “respect for,” safe and inclusive environments, diversity, “research should,” and Métis context. They have been modified within the “Saskatchewan Métis Health Research and Data Governance Principles©” to increase clarity. Principle 2, “respect for” has been changed to “respect” and Principle 5, “research should” has become “research ethics.” Ultimately, the “Saskatchewan Métis Health Research and Data Governance Principles©” aim to foreground Métis rights holders’ interests throughout all stages of the research life cycle, from envisioning research inquiries, to housing data, and enacting knowledge mobilization strategies.

Key differences exist between the “Saskatchewan Métis Health Research and Data Governance Principles©” and OCAP® principles. Unlike the OCAP® principles which call for strict ownership and control of data and research processes, the Métis principles prioritize relationships, respect, and reciprocity in research environments to ensure that productive, respectful, and safe relationships are at the core of all Métis research (Tait and Henry 2022). However, similar to OCAP® principles, the principles of Métis research require that all research are informed by Métis protocols, knowledge, and values (Tait and Henry 2022). Below we have defined the specific meanings of each of the six principles.

### Reciprocal relationships

This principle encourages the establishment of mutually beneficial relationships through meaningful engagement between Métis rights holders and research partners. It clarifies that engagement/involvement can and will look different across community/organizational contexts, but that it must remain mutually agreed upon by the rights holder and researcher. Reciprocal relationships facilitate a collaborative approach to projects that prioritizes Métis involvement in developing research priorities, methodologies, data governance, and knowledge mobilization strategies, specifically through participation from Métis individuals with lived experience. The principle encourages research partners to consult with rights holders about their history, present circumstances, and culture to ensure the research is facilitating localized approaches to knowledge creation. Finally, this principle outlines that many Métis communities, locals, and organizations lack the internal capacity to participate in research; therefore, part of building reciprocal relationships is supporting partners with the financial and human resources to participate meaningfully in research.

## Respect

The second principle highlights the importance of respect for Métis approaches to research, including practices, protocols, individual and collective autonomy, identity, and values. It notes that researchers should consult Métis communities about their preferred practices or protocols, as they vary from community to community and individual to individual across a “wide-ranging contemporary to traditional continuum.”

## Safe and inclusive environments

This principle calls for a culturally safe research environment that is inclusive of diverse Métis voices and perspectives across both social and geographical contexts, and that is guided by the appropriate provincial, regional, and/or local community people. It explains that cultural safety is relative to each Métis rights holder, and that cultural concepts and ceremonies may only be incorporated when local individuals identify them as important to the research process.

## Diversity

Building from the principles of safe and inclusive environments, diversity highlights the inter-Métis perspectives and lived experiences. It emphasizes that grouping “the Métis” into one category fails to recognize the diversity of identities, lifestyles, cultural beliefs, and practices of Métis people across local, regional, and national contexts that have been exacerbated due to colonialism.

## Research ethics

This principle refers to the ethical purpose of a research project and its processes. That is, in order to be considered ethical, the project must acknowledge and protect Métis knowledge and sovereignty and be beneficial and accountable to community interests.

## Métis context

The final principle, Métis context, acknowledges the importance of the local diverse histories of Métis communities/organizations and requires the researchers to familiarize themselves with the place and the people they are collaborating with. The principle encourages research and institutions to undertake this work prior to attempting to form research partnerships.

## Considerations

While the global acceptance of open data has highlighted the need for accessibility, one of the primary challenges for Indigenous nations is structural issues such as capacity to engage in Indigenous data governance (Hahmann and Kumar 2022, Gabel 2025). Such initiatives require data storage and security, professional expertise, as well as expensive technology and software that are embedded in Indigenous collective and individual rights (Leone 2021). This need highlights the importance of sustainable funding models and infrastructure to support data storage. To address capacity and infrastructure, Métis peoples and nations should (i) be involved at every step of the research lifecycle which is equally as beneficial for Métis rights holders as owning and controlling data and (ii) recognize that storing data might not be the most pragmatic option for Métis Nations at this point in time.

## Conclusion

Indigenous data rights, sovereignty, and governance in Canada have largely been framed through a First Nations perspective. This approach overlooks the need for distinction-based, localized methods of research and data governance for Métis peoples. The absence of Métis-specific research guidelines has contributed to the underrepresentation of Métis realities in research, often leading to assumptions that their needs align with those of other Indigenous communities. This has left Métis, one of the three constitutionally recognized Indigenous peoples in Canada, behind in research and policy initiatives, and dependent on data frameworks that do not fully meet their unique needs. The “Saskatchewan Métis Health Research and Data Governance Principles©” aim to address this gap by recognizing the distinct history, cultures, and values of Métis peoples, thereby advancing Métis research and data sovereignty. While these principles share similarities with the First Nations Principles of OCAP®, they also reflect Métis-specific priorities, including the capacity building and meaningful engagement with Métis rights holders throughout the research process. Ultimately, the “Saskatchewan Métis Health Research and Data Governance Principles©” provide an essential framework for guiding research in Métis health and well-being, ensuring that Métis perspectives and values are embedded in every stage of the research lifecycle.

## Data Availability

Not applicable.
